# A paradigm shift in neutrophil adverse event grading: What now?

**DOI:** 10.1002/hem3.70266

**Published:** 2025-12-28

**Authors:** Lauren E. Merz

**Affiliations:** ^1^ Division of Hematology/Oncology, Department of Medicine University of Michigan Ann Arbor Michigan United States

The Common Terminology Criteria for Adverse Events (CTCAE) have been used by most clinical trials globally since the 1980s to record the incidence and severity of toxicities associated with systemic anticancer therapy.^1^ The most recent update (v6) was released in the summer of 2025 with planned implementation for clinical trials on January 1, 2026. One of the notable changes includes a significant update to the neutrophil grading criteria, which essentially translates neutrophil count grade up by one level, where absolute neutrophil count (ANC) < 1500–1000/µL is now Grade 1 (previously Grade 2), and Grade 4 is now ANC < 100/µL (Table 1).

**Table 1:** Comparison of CTCAE v5 and CTCAE v6 neutrophil count grading criteria.

	CTCAE v5 (2017)	CTCAE v6 (2025)
Grade 1	LLN–1500/µL	<1500–1000/µL
Grade 2	<1500–1000/µL	<1000–500/µL
Grade 3	<1000–500/µL	<500–100/µL
Grade 4	<500/µL	<100/µL

*Note*: The shading highlights similar ANC levels between CTCAE v5 and v6.

Abbreviations: CTCAE, common terminology criteria for adverse events; LLN, lower limit of normal.

The potential reasons for this timely and necessary CTCAE neutrophil count grade update were outlined in a previous HemaTopic and summarized in Figure 1.^2^ The change is more inclusive for people with the Duffy null variant—a genetic variant commonly found in people with genetic ancestry from Sub‐Saharan Africa or the Arabian Peninsula resulting in lower ANC without increased risk for infection.^3^ Additionally, this update is likely an acknowledgment that many modern therapies are targeted and the mechanism of cytotoxicity (if any) is different than older therapies.^2^ It also integrates decades of observational data on neutrophil levels correlated with the degree of concern for febrile neutropenia or serious infectious complications.^4^ However, we now must grapple with the implications of a change to a key adverse event criterion for ongoing and future clinical trials.

**Figure 1:**
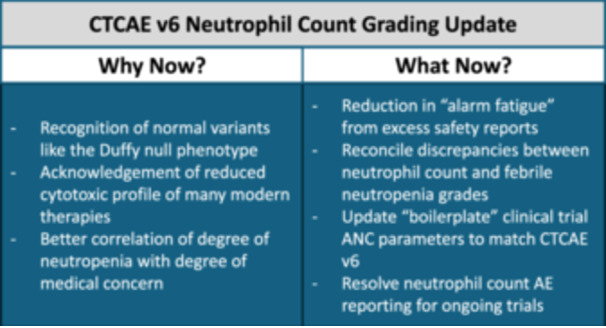
**Summary of why the Common Terminology Criteria for Adverse Events (CTCAE) may have updated the neutrophil count grading as well as ways in which these updates will likely impact ongoing and future clinical trials.** AE, adverse event; ANC, absolute neutrophil count.

Encouragingly, these CTCAE changes are in line with recent recommendations from the coalition for reducing bureaucracy in clinical trials (RBinCT).^5^ This cross‐disciplinary group involving patient advocates and medical societies has identified the need to streamline the communication of safety reports communicated to investigators to reduce “alarm fatigue” and ensure that investigators react quickly to relevant reports.^5^ The updated neutrophil count grading criteria will help with this goal. For example, if safety reports are routinely generated for a Grade 3 or 4 neutrophil count, this will now trigger a report when ANC is <500/µL by CTCAE v6. A neutrophil count of 300/µL (Grade 3 by CTCAE v6) is much more concerning and urgently actionable (e.g., granulocyte colony‐stimulating factor administration) than an ANC of 900/µL (Grade 3 by CTCAE v1–5). This will likely reduce excessive safety reports, which will help investigators respond quickly and appropriately to medically relevant concerns.

One potential issue to reconcile is the disconnect between the definition of febrile neutropenia and updated neutrophil count grading. In both CTCAE v5 and CTCAE v6, febrile neutropenia is defined as an ANC < 1000 with a temperature of >38.3°C or sustained at >38°C for more than an hour. By CTCAE v1–5, this serious complication was paired with a Grade 3 neutrophil count. Grade 3 events are indeed intended to indicate a severe or medically significant event. However, an ANC < 1000–500/µL is now a Grade 2 event by CTCAE v6—representing a moderate risk event with noninvasive interventions potentially required. Is it still accurate to label a patient with an ANC of 800/µL (Grade 2) with a fever to 38.4°C as febrile neutropenia? Or would it be more accurate to indicate that this patient has a Grade 2 neutrophil count with a Grade 1 fever? The urgency and interventions needed for these two scenarios are dramatically different. Furthermore, many studies and societies (including the American Society of Clinical Oncology) already use an ANC < 500/µL to define febrile neutropenia rather than an ANC < 1000/µL.^6^, ^7^ This is an area that needs to be reconciled. Given the available data supporting minimal infectious risk when ANC is >500/µL^4^, ^7^ and the appropriately updated neutrophil count grading in CTCAE v6, I strongly recommend updating the CTCAE febrile neutropenia criteria to an ANC of <500 with a temperature of >38.3°C or sustained at ≥38°C for more than an hour in the next iteration.

Additionally, many clinical trials have linked CTCAE neutrophil count grading levels to clinical trial parameters, such as eligibility criteria or criteria for medication dose modification. Although trials should be personalized for the regimen tested and as inclusive as possible, many are not, instead following “boilerplate” eligibility criteria.^8^ For example, the boilerplate ANC threshold for clinical trial eligibility is often ANC ≥ 1500/µL.^9^ This indicates that patients cannot have a Grade 2 neutrophil count or worse as defined by CTCAE v1–5 on entering the trial. However, CTCAE v6 now defines Grade 2 neutrophil counts as <1000–500/µL. Will the boilerplate ANC eligibility criteria be updated to an ANC ≥ 1000/µL to enter a trial, reflecting the exclusion of patients with Grade 2 neutrophil counts or worse? Or will it remain at ANC ≥ 1500/µL and thus exclude patients with any CTCAE v6 grade of neutrophil count? Again, the gold standard is for clinical trial eligibility criteria to be maximally inclusive and tailored to safety signals seen in Phase 1 and 2 testing.^8^, ^10^ However, as we strive towards that ideal goal, I recommend updating the “standard” ANC eligibility criteria to ≥1000/µL to reflect CTCAE v6 criteria. Regulatory review boards, principal investigators, and collaborators should be encouraged and empowered to request evidence‐based justification for any ANC eligibility threshold set above 1000/µL.

Furthermore, there are serious questions about how this CTCAE update might impact ongoing clinical trials. The National Cancer Institute (NCI) website states, “Implementation [of CTCAE v6] for…studies is targeted for January 1, 2026. It will apply to newly opened studies only.” However, ongoing studies risk falling out of step with the ubiquitous reporting of neutrophil count adverse events by modern CTCAE grades by the time of publication. This could result in ongoing studies appearing outdated before their time or difficult to compare with other contemporary trials. However, updating clinical trial parameters or adverse event grading mid‐trial is challenging and sub‐optimal. There may be no easy answers for ongoing clinical trials. For ongoing trials that opened during the CTCAE v5 timeframe and close in the CTCAE v6 era, I recommend reporting the proportion of patients that fall within numerical ANC thresholds (i.e., <1000–500/µL), mirroring the CTCAE v6 thresholds, rather than reporting the proportion of patients by grade levels (i.e., Grade 2).

In summary, the CTCAE v6 neutrophil count grading criteria update is a significant change with potentially broad impacts on clinical trial design and adverse event reporting. Although these updates are timely and necessary to reflect modern therapies and ensure maximal inclusivity, this is a major departure from the clinical trial status quo over the past 40+ years. There will certainly be growing pains as clinical trial protocols and the reporting of adverse events are updated. However, I strongly believe that these changes will ensure optimal inclusivity, reduce alarm fatigue, better connect neutrophil count grade to risk of infectious complications, and reflect the cytotoxic realities of most modern systemic anticancer therapies. I eagerly anticipate reviewing clinical trial protocols and publications in the coming years to see how some of these changes may actualize.

## AUTHOR CONTRIBUTIONS

**Lauren E. Merz**: Conceptualization; writing—original draft; writing—review and editing.

## CONFLICT OF INTEREST STATEMENT

Dr. Merz reports receiving personal fees from Johnson&Johnson and 23andMe.

## FUNDING

This research received no funding.

## Data Availability

Data sharing is not applicable to this article as no datasets were generated or analyzed during the current study.
